# Perspectives of family caregivers and nurses on hospital discharge transitional care for Muslim older adults living with COPD: a qualitative study

**DOI:** 10.1186/s12912-024-01943-8

**Published:** 2024-04-24

**Authors:** Latifah Jehloh, Praneed Songwathana, Luppana Kitrungrote, Anne Bourbonnais

**Affiliations:** 1 Faculty of Nursing, Prince of Narathiwas University, Muang, Narathiwat, Thailand; 2 Faculty of Nursing, Prince of Songkla University, Hatyai, Songkhla, Thailand; 3 Faculty of Nursing, Université de Montréal, Montréal, Québec, Canada

**Keywords:** Barriers, Facilitators, Hospital discharge, Muslim, Older adults living with COPD, Transitional care

## Abstract

**Background:**

The increased number of emergency department visits among older adults living with chronic obstructive pulmonary disease reflects the challenges of hospital discharge transition, especially in those from a cultural minority. The barriers and facilitators of this discharge from the perspective of formal and informal care providers, such as nurses and family caregivers, are important to identify to provide effective symptom management and quality of care. The purpose of this study was to describe the barriers and facilitators in caring for Muslim older adults with chronic obstructive pulmonary disease (COPD) during hospital discharge transitional care.

**Methods:**

A descriptive qualitative study was conducted in a hospital of Thailand where Muslim people are a cultural minority. Thirteen family caregivers of Muslim older adults living with COPD and seven nurses were purposively recruited and participated in semi-structured interviews and focus group discussions. Content analysis was used to analyze the data.

**Results:**

Five barriers and three facilitating factors of transitional care for Muslim older adults living with COPD were outlined. Barriers included: (1) lack of knowledge about the causes and management of dyspnea, (2) inadequate discharge preparation, (3) language barrier, (4) discontinuity of care, and (5) COVID-19 epidemic. Facilitators included: (1) the ability to understand Malayu language, (2) the presence of healthcare professionals of the same gender, and (3) the presence of Muslim healthcare providers.

**Conclusion:**

Family caregivers require more supportive care to meet the care needs of Muslim older adults living with COPD. Alternative nurse-based transitional care programs for these older adult caregivers should be developed.

**Supplementary Information:**

The online version contains supplementary material available at 10.1186/s12912-024-01943-8.

## Introduction

Chronic obstructive pulmonary disease (COPD) in older adults has received significant attention worldwide due to its trajectory of gradual health decline with acute exacerbations and at risk of mortality due to frailty [1]. In 2019, COPD was estimated by WHO to be the third leading cause of death in the world [2], with an increasing prevalence, particularly among older adults [3]. In the upcoming decades, it is anticipated that the prevalence of COPD will continue to rise in Asian nations along with disease mortality. The epidemic of tobacco uses and air pollution in those countries are the main causes of this increase. For example, in Thailand, the prevalence of COPD in older adults is increasing [4], with 14% of people aged 65 years or older having the disease compared to 9.9% of younger people [5].

Older adults with COPD have high symptom burden, healthcare utilization, and mortality. Many of those patients’ needs are unmet as well as those of their family caregivers [6]. Nearly half of older adults with COPD experienced at least one exacerbation per year that required emergency department (ED) care [7]. This causes them to transition often between their home and the hospital. Those care transitions following an ED visit or hospital discharge are frequently disorganized and poorly managed leading to further increase in ED visit rate, adverse medical events, and caregiver burden [8]. Transitional care refers to various steps intended to guarantee that patients’ care is coordinated and continues while they move between different facilities or levels of care [9]. It enables their safe and prompt transition across care settings [10]. Adequate transitional care for older adults is essential because it can minimize medication errors, and adverse drug events, and help prevent lack of timely follow-up care management, and unnecessary ED visits [11]. Therefore, successful hospital-initiated transitional care programs should incorporate a “bridging” strategy with pre- and post-discharge interventions, and a dedicated transitions provider involved at various points in time. Multicomponent strategies to enhance an effective transitional care during and after hospital discharge (from ED or a care unit) is required. These include patient engagement, use of a dedicated transition provider, and facilitation of communication with outpatient providers which require time and resources [12]. In the context of COPD, the transition is important to improve symptom management skills at home and requires the support of three main key persons; older adult patients, family caregivers, and healthcare professionals. To promote an adequate transition, it is often recommended that patients with COPD actively participate in the management of their condition to achieve better health outcomes [13].

However, older adults with COPD can face multiple limitations in their activities of daily living. As a result, patients may be forced to depend more on their family caregiver. Those caregivers can be critical to provide both practical assistance and emotional support [14]. They can assist older people by monitoring prescription medication use and doctor appointments, helping with personal care, offering emotional support, and accompanying them to the hospital [15].

Besides family caregivers’ support for older adults, health professionals have also been reported as important resources in the older adult’s ability to cope with COPD [16]. The quality of life for COPD patients may be improved by expanding the role of primary and hospital nurses. Using nurse-led care models can improve clinical results and patient satisfaction [17]. Home visits and remote management with telemedicine at discharge time by hospital and community nurses can also support transitional care [18]. When nurses care for COPD patients, daily disease management results are improved for both COPD patients and their family caregivers [17]. Support during care transitions between clinicians or settings are then necessary.

## Background

A few studies focused on the facilitators and barriers of these transitional care for older adults with COPD following an ED visit or hospitalization. A study in the Netherlands [19] conducted a mixed method study to explore the perspectives of COPD patients, family caregivers, and health care providers. It was found that three possible influencing factors to promote and implement self-management in daily practice as a transitional care strategy were patient competence with COPD, healthcare provider competence, and organization collaboration. Another qualitative study conducted in rural Nepal [20], factors related to the patient, the clinician, and the service had an impact on the self-management of COPD patients during transitional care. People with COPD found to have a low understanding of the disease and treatments and had insufficient family support. Limited rural resources in health services were identified such as a lack of COPD teaching tools, inadequate primary level clinician’s competence, and inadequate infrastructure. Language barriers from a cultural minority were concerned [20].

Therefore, being a member of a cultural minority, especially if speaking another language, can be a barrier to care for older adults with COPD. There is a scarcity of research on the factors that help and hinder people from providing care for older adults with COPD, particularly if they are part of a cultural minority. There are about 70 million population in Thailand in 2022 [21], most of the population is Buddhist, and communicate using the Thai language [22]. But there is 7 million people who are Muslim and are considered a cultural minority [23]. Most Muslims of the country live in the three southernmost provinces of Thailand: Yala, Pattani, and Narathiwat. 90% of the people in these provinces are Muslim and speak the Malayu language. The most common chief complaint presented by older adults with disease-related ED visits was dyspnea from COPD (Paksopis et al., 2019; Song et al., 2016). Similarly, the highest numbers of older adults visiting the ED at one of the provincial hospitals in southern Thai-Malaysian border areas due to dyspnea from COPD as reported between 2016 and 2021 were 488, 669, 513, 484, 453 and 364 respectively [24]. The Muslim cultural belief can have an impact on the health and illness as it is influenced by the Islamic religion. For example, there have specificities regarding diet and medications which must be halal products. Diet and medication prescription during Ramadan need to be adjusted because they cannot consume anything during the day of that month [25]. To offer culturally competent care, it is crucial to study the unique cultural perspectives on the factors influencing transitional care for older adults with COPD since they differ in terms of their religious beliefs, personal life, and approaches to health care.

Nurses are primary health care providers (HCPs) in transitional care who collaborate with other HCPs to assess patients’ biological, psychological, social, and emotional needs; offer community care services, such as home health and outpatient care; and identify obstacles to providing follow-up care and services. Therefore, their attitudes and perceptions may lead to the safety and quality of transitions [26]. However, there is a lack of knowledge on the factors (facilitators and barriers) influencing this type of care from the perspective of family caregivers and nurses. This population has additional challenges because they care for older people and a Muslim minority with cultural aspects that are intertwined in the situation. A qualitative research approach is useful to describe such factors with the intention of enriching recommendations for better practice [27]. This study is the first phase of a larger intervention program of research. It aimed to provide a thorough description of family caregivers and nurses perspectives on facilitators and barriers to COPD symptoms management during transitional care following an emergency department visit or hospitalization of a cultural minority, Muslim older adults, living with COPD in southern Thailand.

### Purpose

This study aimed to explore the barriers and facilitating factors in caring of Thai Muslim older adults with COPD regarding hospital discharge transitional care either an emergency department or medical unit from the perspective of nurses and family caregivers.

### Research question

What are the barriers and facilitators in caring for Muslim older adults with COPD?

## Methods

The concept of hospital discharge transitional care based on the Naylor’s model. The main aim of transitional care strategies, according to this concept, is to prevent avoidable readmissions and unfavorable health effects after a hospital discharge. The hospital discharge transitional care programs are often targeted at chronically ill adults or patients who are at risk of poor post-discharge results, as well as their informal caregivers, such as family and friends [28]. A descriptive qualitative study was employed in this study. This research design is often used to describe phenomena as paradigm, for example, a constructivism perspective related to nursing and health care [29]. It is primarily concerned with identifying the who, what, and where of experiences about a complex phenomenon [30]. It is consistent with our aim to explore and generate a rich understanding of family caregivers and nurses’ perspectives on caring of people living with COPD after patient discharge to home. Furthermore, it is the research design of choice when a straightforward explanation of a phenomenon is needed to develop interventions [31].

### Study setting and recruitment

The provincial hospital in the Narathiwat Province located in the southern Thailand was selected as the study’s setting. It is regarded as the one of the highest number of older adults visiting the ED because of COPD since 2016 to date. In this hospital, Muslim older adults with COPD are hospitalized on two departments requiring transitional care either the medical department or the emergency department.

When patients are discharged from this hospital, the Community Health Centers take over the transitional care by visiting older adults with COPD within two weeks of their discharge from the hospital. The Community Health Centers are in the same care network as the hospital and are the primary care units in a subdistrict (also called Tambon) or village. Each one serves a population of 1,000 to 5,000 people. They are registered nurses, and the equivalent of a licensed practical nurse. The services include patient evaluation, care planning and, home visits as well as supervise of village health volunteers. Those volunteers receive training to conduct routine home visits for patients, assist with activities of daily living, provide basic healthcare services, offer spiritual support to patients and family members, and monitor the home environment [30]. Four community health center nurses participated in our study as they offered transitional care, to older adults with COPD after they were discharged by the hospital. As such, the hospital, patient’s home, and Community Health Centers served as this study’s settings.

### Inclusion criteria

We purposively recruited 13 family caregivers (FG) of Muslim older adults living with COPD and seven nurses who offered transitional care to Muslim older adults living with COPD, either at the hospital or in the community.

FG of an older Muslim adult living with COPD were recruited with the help of hospital nurses. They were included if they had a close relationship with a person aged 60 years or older. They could be a spouse, daughter, or son who provide care for the older adult with COPD both in the hospital and at home after being discharged. They had to be able to communicate well in Thai or Malayu language. Although the interview was conducted at the participant’s home, they were asked of their willingness to participate while the older adult was still in the hospital. In addition, nurses who offered transitional care in the ED, a medical department, and/ or with a community health center that cared for Muslim older adults living with COPD were asked and invited to participate in the study. They were willing to share their experience and have the time.

The number of participants in qualitative studies usually requires at least 12 participants to reach data saturation on a topic [32]. Therefore, 13 family caregivers who provided direct care to older adult patients with COPD were recruited for the study. In addition, seven nurses were purposively recruited to participate in focus group discussions. The number of participants was adequate when no new data were discovered and the researchers had a good understanding of the participants’ perspectives.

### Data collection

Data were collected from nurses through focus group discussions (FGDs), and from family caregivers through individual semi-structured interviews by the first author. The interview guide in this study was developed by the researchers which validated by three experts. The two separated interview guides were used, one for family caregivers and another for nurses. Both guides were developed by the researchers and validated by three experts consisting of two nursing lecturers, and one nurse. These guides focused on their perspectives of barriers and facilitators of symptom management of Muslim older adults living with COPD and their transitional care needs. An example of a question for the family caregivers is: “What are the barriers for Muslim older adults living with COPD to manage their symptoms at home after being discharged from the hospital?”. An example of a question for the nurse is: “What kind of discharge preparation for COPD symptom management do you offer Muslim older adults before they are discharged?”. Family caregivers were interviewed individually in their homes. FGDs with nurses were held online via Zoom because of the COVID-19 pandemic. It is recommended that the number of participants in a focus group should be a minimum of 4 and a maximum of 12 participants per group [33]. Therefore, we conducted the FGD twice with the same seven nurses to validate data. Both the FGD and the semi-structured interview were recorded on digital audiotape with the participants’ permission. The FGD lasted no more than two hours, and each semi-structured interview lasted about 40 to 45 min. Existing documents were also collected, particularly discharge planning, nurse guidelines, and the service plan related to the care of older adults with COPD during their transition from hospital to home were examined.

### Data analysis

A content analysis, as proposed by Schreier in 2012 [34], was performed. The data from interviews and focus groups were transcribed verbatim. The transcriptions were read line by line several times while these descriptions were reviewed. The analysis was conducted in five stages. The first stage was to create a template for the analysis, which included a memo sheet and coding units. Second, all of the data including field notes, observation recording forms, and interviews and focus group transcriptions, were gathered together and examined several times in order to understand the informants’ perspectives and the connection between the data and the research goal. Third, the main ideas expressed by the informants were inductively coded. Fourth, the core subject and its subthemes were evaluated, subjects were compared, and tables were created. The codes were arranged in tables to show the similarities and differences in the codes. Finally, the main theme and subthemes were formed based on this coding organization. We specifically highlighted facilitators and barriers that are typical of Muslim older adults living with COPD from the perspective of nurses and family members. The results of the content analysis were confirmed by coresearchers through discussion.

### Rigor and reflexivity

Prolonged involvement, and member verification are techniques to ensure credibility in this study [35]. The verbatim transcriptions of the audiotape records and memos were kept for an audit trail. In addition, the results were eventually given to the participants in different meetings to allow the researcher and participants to view the data from distinct perspectives and improve data analysis. Confirmability was achieved through the researchers being aware of subjectivity by using a member checking and peer review.

### Ethical considerations

Approval was obtained from the Research Ethics Review Committee of the Faculty of Nursing, XX University (Approval no. IRB 2021-LL-Nur023). Participants were provided with written information explaining the purpose of the study, procedures, and plans to maintain confidentiality. Participants were also informed of their right to withdraw from the study at any time without interfering with health care service. Both verbal and written consent were given for all participants prior to the start of this study.

## Results

This study included thirteen family caregivers (ten women and three men). Their ages ranged from 25 to 80 years old. The relationships between the family caregivers and the older adults they cared for were: 5 daughters, 4 spouses, and 4 sons. Most of them (38.5%) had completed primary school. All family caregivers lived with the older adults. Nurses (*n* = 7) were all female and ranged in age from 42 to 55 years old. More than half had a bachelor’s degree, and three had a master’s degree. They had 20 to 32 years of experience in caring for older adults with COPD. They worked in the medical ward (*n* = 2), ED (*n* = 1), or community health centers (*n* = 4).

Our findings identified eight themes describing barriers and facilitators to transitional care as shown in Table 1. Some themes were mentioned by both family caregivers and nurses and other only by family members. The themes on barriers mentioned by both nurses and family caregivers related to insufficient discharge planning, discontinuity of care, and the COVID-19 pandemic’s effects. The two additional themes that were highlighted by family caregivers: language barriers and a lack of knowledge about the causes and treatment of dyspnea symptoms. Additionally, family members identified facilitators which nurses did not.

**Table 1:** Summary of themes describing the barriers and facilitators to transitional care

Barriers to transitional care	Facilitators of transitional care
Lack of knowledge about the causes of dyspnea and its management (family caregivers)	Ability to understand Malayu language (family caregivers)
Inadequate discharge preparation (family caregivers and nurses)	Presence of health professionals of the same gender (family caregivers)
Miscommunication due to a language barrier (family caregivers)	Presence of culturally competent health professionals (family caregivers)
Discontinuity of care delivered after discharge (family caregivers and nurses)	
Impact of the COVID-19 pandemic (family caregivers and nurses)	

### Barriers related to symptom management during hospital discharge transitional care

The findings revealed five themes describing barriers related to symptom management after being discharged to home from the perspectives of family members and nurses.

1. Lack of knowledge about the causes of dyspnea and its management

The majority of Muslim older adults with COPD in this study relied on family members for physical, psychological, emotional, and spiritual support. Most of them had only completed primary school, so they were not very familiar with the Thai language. Therefore, they were ignorant about how to treat the symptom of dyspnea and felt unprepared to offer care. They were more likely to bring the older adults they cared for to visit the ED because they were afraid when their relatives were out of breath. These family caregivers wanted more guidance in understanding the condition and knowing whom to call when their relative developed dyspnea at home. Family caregivers also perceived that dyspnea was associated with a rapid decline in the health which increased the risk of death. As family caregivers mentioned:

It was terrifying when he developed dyspnea. I was nervous and scared, and I was not sure what I should do. (FG 13)

I did not know what are the causes of dyspnea and how to manage when he had shortness of breath. (FG 2)

Regarding acute exacerbations and the patient’s decline, family caregivers sometimes felt uncertain about the trajectory of the disease. They also wanted to help better manage symptoms like breathlessness. As a result, most of family caregivers found it extremely challenging to support their relative manage symptoms at home.

2. Inadequate discharge preparation

Nurses who participated in the study mentioned that when Muslim older adults living with COPD were discharged from the medical ward, they were given a brief education using the D-method guideline. This guideline stands for D: disease, M: medication, E: environment, T: treatment, H: health, O: outpatient appointment, D: diet. However, family caregivers mentioned that nurses were sometimes pressed for time due to other responsibilities. Their discharge preparation interventions for the older adults were general rather than specific to their needs. The effectiveness of providing education prior to discharge depended on who provide the education and how busy a time of the day it was. In addition, if the older adult was newly diagnosed with COPD, the pharmacist came to teach them how to use an inhaler once. However, only the ward nurse came to educate them before they were discharged if they returned for a subsequent treatment.

When Muslim older adults with COPD were discharged from the ED, their discharge may have been unplanned because there was no time for discussion and education. In the ED, all discharge forms were designed to be quick and easy for health professionals. To save time, nurses were not required to answer lengthy questions. However, using these discharge forms with older adults with COPD before discharging was not considered as a good idea because of the time required to answer questions. As both family caregivers and nurses stated this:

Nurses were very busy all the time. They were hurrying up to give me education especially in the ED. (FG 6)

Nurses educated me only one time before discharging. (FG 3)

At ED, most forms were designed to be filled out quickly before discharge. To save time, nurses were not required to answer lengthy queries. In addition, using these forms for older adults with COPD before discharging may need time to respond and ask questions. (Nurse 1 from ED)

At the medical ward, Muslim older adults with COPD were discharged by using the D-METHOD form. The effectiveness of providing education prior to discharge was dependent on who was providing the instruction and the busy time of the day. If the older adults were diagnosed with COPD for the first time, the pharmacist had come to teach them how to inhale the drug once, but if they returned for a second or subsequent treatment, only the ward nurses had come to educate them before they were discharged. (Nurse 2 from Med ward)

3. Miscommunication due to language barrier

It was found that some Muslim older adults with COPD and family caregivers were unable to communicate in Thai. They usually use the Malayu language in their daily life. Since health professionals were unable to communicate with them in Malayu, this added limitations during the discharge process. It seems to be more difficult to teach them about the health of the older adults and care needs using the unspoken language. Furthermore, there were numerous challenges in explaining, educating and teaching back because older adults and their family caregivers were less likely to communicate or understand all in Thai. This is reflected by both family caregivers and nurses:

Nurses always talked to me in Thai. This made me unsure about the information because I could not understand them some words. (FG 3)

I could not be able to understand 100% if nurses talked or communicated to me in Thai language. (FG 2)

In teaching patients, we were unable to communicate in Malayu since the older adults and family caregivers were unable to communicate in Thai. (Nurse 2 Med ward)

4. Discontinuity of care delivered after discharge

According to discharge process, nurses from the medical ward were required to send discharge information to the community nurses as part of the Continuum of Care (COC) program to conduct a home visit within 14 days of discharge. However, the care plan for Muslim older adults with COPD or patient information was not sent to community nurses after discharged from the ED, resulting in a lack of continuity of care with home visits. Therefore, sending patient information about specific COPD care (e.g., equipment demonstration, nutrition support, medication review) was mentioned as something that could help community nurses. So, several older adults with COPD did not receive continuity of care from community nurses after discharge. As both family caregivers and nurses mentioned:

The community nurse visited my husband when he was admitted to the ward, however, after visiting the ED last week, no nurse came. (FG 7)

There was no nurse visiting me after hospital discharge at home. (FG 6)

Every COPD patient who was discharged from medical ward was sent to community nurses through the COC team for continued care at home before discharging patients, except when patients only visited the ED without being admitted to the hospital, in which case the information would not be passed through this system. (Nurse 1, community health center)

5. Impact of the COVID-19 pandemic

During our study, some community nurses were quarantined because they had been exposed to COVID-19. There were no enough nurses available on duty due to an outbreak of COVID-19 in the village. They were unable to make routine home visits after patients were discharged, as one of the family caregivers explained:

In the midst of the COVID-19 outbreak, the nurse did not visit us as usual. (FG 10)

When these weekly home visits by community nurses took place, it was considered by family members to be a good method for Muslim older adults living with COPD to manage their symptoms properly at home. In addition, COVID-19 resulted in a few weeks of lockdown in some communities, as one caregiver and nurse mentioned:

The city was locked down right now because of COVID, making it exceedingly impossible for us and those outside to move in and move out of the city. (FG 13)

Under normal conditions, community nurses were required to visit all patients within 14 days after discharge. Due to an outbreak of COVID-19 epidemic in the village, some community nurses were quarantined due to contaminated with COVID-19. Since there was the shortage of nurses on duty, therefore, we were unable to visit patients’ homes as plan. (Nurse 4, community health center)

As a result, it was extremely challenging for nurses to conduct home visits. Furthermore, acute exacerbations of COPD in older adults were also associated with COVID-19. Thus, the COVID-19 pandemic had numerous effects on how older adults with COPD and their caregivers managed their symptoms at home. For instance, they were unable to receive home visits or health education after hospital discharge, they lost contact with people outside the village, and found it extremely difficult to travel to the hospital for monthly medical appointments.

### Facilitators related to symptom management during hospital to home discharge transition

The participants revealed what family caregivers and nurses found to be helpful in managing the symptoms of Muslim older adults with COPD during transitioning from the hospital to their home. Three themes describing those facilitators were identified.

1. Ability to understand Malayu language

Communication and education between health care professionals and family caregivers depended heavily on language. Because many family caregivers were unable to converse in Thai as mentioned before, family caregivers felt safe and trusted health professionals when they spoke Malayu. They found it helpful to discuss their needs and difficulties in their native language. For instance, a caregiver stated:

When I communicated with health professionals who spoke the same language as me, I felt safe. I struggled to communicate in Thai. (FG 1)

I can fully understand when nurses talked to me in Malayu language. (FG 12)

They sought out health professionals who spoke Malayu to ask medical-related questions and learn about symptom management. As such, language was an important facilitator during transitional care.

2. Presence of healthcare professionals of the same gender

According to the participants, same-gender health professionals could be approached, spoken to, or discussed with confidence because they could make eye contact without being shy. Participants believed that interactions with someone of the opposite gender could be immoral. As a family caregiver and for their relative living with COPD, they found it extremely comforting to be cared for by a person who was of the same gender and that facilitated transitional care. Some family caregivers who adhered to very strict religious doctrine demanded that only health professionals of the same gender provide care for their relative. They sometimes refused to receive care from a doctor of the opposite gender, unless they were no other option. This search for a same gender doctor was based on the belief that it is sinful to make eye contact or have physical contact with someone of the opposing gender. As a result, they wish to ensure their relative would not be in this situation. As some caregivers stated:

I felt comfortable making eye contact when discussing or communicating with health professionals who are of the same sex as me. (FG 2)

My mother was very old, she felt discomfort telling her symptom with men doctor. (FG 5)

3. Presence of culturally competent health professionals

For the participants, understanding Muslim spiritual and cultural values was necessary to provide care for Muslim older adults in a health care context. In this study, Muslim family caregivers were extremely devout in their religious observance. Aspects of daily life, such as diet, self-care, gender interaction, beliefs, and personal life, were incorporated as elements of Islamic law. They explained that the Muslim population is made up of a wide variety of ethnic groups. As such, they mentioned similarities and differences in terms of religious beliefs concerning health. Due to the cultural beliefs and the need for cultural competence when dealing with Muslim older adults, many non-Muslim health professionals encountered challenges when caring for Muslim patients and their family. Decision-making, health habits, and healthcare utilization were all influenced by the Islamic faith. So, family caregivers preferred to seek care from Muslim health professionals to comply health examinations in a way that respected beliefs, and ability to adapt health and dietary advice in accordance with Islamic law. When Muslim health professionals interacted with family caregivers using an Islamic method, they trusted and followed the instruction or education. This facilitated the health education on symptom control prior to hospital discharge. As family caregiver stated:

I felt very happy to talk and ask questions to Muslim health professionals because they educated me by using Islamic principles. (FG 6)

Muslim doctor knows a lot about Islamic rules and appropriately approached with Muslim patients. (FG 9)

## Discussion

The findings revealed that current services for Muslim older adults living with COPD were still limited in terms of informing and responding to the needs of family caregivers. After patients were discharged from hospital, family caregivers need some support with continuing care and a regular follow-up. In our study, the discharge preparation provided by health professionals was insufficient due to lack of time and language barriers. Furthermore, despite a well-functioning home visiting system, home visits after discharge are difficult, especially after ED visit, and were limited during the COVID-19 pandemic.

Lack knowledge regarding the causes of dyspnea and its management is important. This finding is consistent with previous studies that more than half of the caregivers of patients with advanced COPD wanted more support. By understanding what to expect in the future, and more than one third desired more assistance in comprehending the illness, knowing who to contact when worried, and coping with their feelings and concerns are required [36]. In addition, the unclear knowledge regarding the potential course of the patient’s health decline, anxious about the future, and afraid of acute exacerbations can lead family caregivers to be unreadiness for discharge [36]. Similar to a previous study [37] which addressed the support need in dealing with symptoms management like breathlessness and providing information related to available health care resources.

The lack of knowledge about disease and symptom management skills among the family caregiver of this study seems to be related to inadequate discharge planning and discontinuation of care system after discharge. The findings were addressed by both health professionals and family caregivers which is consistent with prior study from the viewpoints of family caregivers [38]. Most family caregivers feel unprepared to provide care because they receive little instructions on how to manage patient symptoms which is consistent with earlier ones [39]. Additionally, dyspnea was a serious symptom perceived by family caregivers. They explained that if left untreated, it could lead to the death of older adults with COPD. As a result, they found it very challenging to manage dyspnea.

In addition, cultural issues especially language was identified as a difficulty in caring for older adults with COPD by both nurses and family caregivers’ perspectives. Thai language is often used by health professionals, while family caregivers usually speak in Malayu. It was quite difficult for both to communicate and obtain more time in health education related to specific disease. This is similar to a previous study [40] that language barriers in healthcare lead to miscommunication between health professionals and patients, making it difficult to provide high-quality healthcare, to preserve patient safety, and to achieve high levels of patient satisfaction. The language barriers or a communication gap between the family caregiver and the health professionals could also increase the healthcare services use and the likelihood of an ED visit [41]. Our study shows that this is an important concern for cultural minority patients and family members. The importance to communicate in the same language would be quite smoothly and had a complete understanding when discuss about disease and its treatment as well as the progression of the disease before discharge. Muslim family caregivers felt safe and opened-mind when health care providers could speak with them in Malayu language. In addition, understanding patients well by communicating in the same language would create a trust and relationship which encourage their participation [42].

Participants also identified that caring for older adults with COPD during the COVID-19 pandemic presented greater difficulties. The COVID-19 pandemic overwhelmed healthcare systems including home health care. Some of the difficulties may cause by the COVID-19 crisis include: (1) a lower volume of home health care referrals, and (2) shortages and costs of personal protective equipment (PPE) and testing [42]. As a result, health professionals were unable to visit patients at home after discharge as usual. This was also the case in our study, where the health community centers were understaffed and there were restrictions on who could enter and exit the village because of COVID-19 outbreak. As a result, it was extremely difficult to visit older adults with COPD at home after they were discharged from the hospital during the pandemic. Patients with COPD were unable to receive care in part because they and their family caregivers stay away from hospitals, even during acute exacerbations.

Furthermore, our results highlight that caring for older adults with a chronic illness such as COPD from a minority culture with different and strong beliefs is challenging. Islam is a religion that has detailed rules and regulations for daily interactions and health-related decisions [43]. In our study, family caregivers believed in God and had a strong religious commitment. As such, they preferred nurses who were Muslim and of the same gender to reduce some barriers and gain more comfortable. Muslim people often consider their religion when making decisions about health care [44]. Some Muslim women who consider themselves as very religious only want same gender professionals [45]. As a result, family caregivers find it easier to understand and build trust when discussing the demands and challenges of caring for older adults with COPD after discharge with Muslim health professionals of the same gender.

Nurses could not identify appropriate approach, language, or content for Muslim older adults with COPD. Family caregivers were then the ones who outlined all facilitators of transitional care for these individuals. As a result, all of them thought it would be better to allow the researchers learnt from family caregivers directly about facilitators of transitional care that will deepen the understanding regarding this topic.

### Strengths and limitations of the work

Our findings are based on the first author’s immersion in the research setting to collect and analyze the data, resulting in their emergence from a natural context. The results give an important description of the thoughts, attitudes, and beliefs of nurses and Muslim family caregivers in southern Thailand. However, there are some limitations to this study. The main one is that this research was conducted during the COVID-19 pandemic. To prevent the spread of the disease, videoconferencing had to be used. It was sometimes more difficult to interact and control the flow of conversation with the participants. In addition, the perspectives of older adults were not triangulated with those of their family members and nurses. This decision was made to avoid adding to the burden of older adults living with COPD who had just been discharged from the hospital. Therefore, future studies could identify means to include them directly.

### Recommendations for further research

Explore alternative approaches to support the care of older adults with COPD and reduce ED visits while improving dyspnea symptom management at home is necessary. The results of this study were perspectives of both nurses and family caregivers. Future studies should be conducted and included older adults with COPD themselves. Interventions that address the cultural aspects of transitional care should also be developed and evaluated.

### Implications for policy and practice

The study’s findings demonstrated the significance of prior information and effective communication in enhancing nurses’ and family caregivers’ comprehension of the illness. Speaking in the local language would therefore be a wise move to enhance comprehension and dialogue. As a result, the study’s result will influence how to provide education, continuity of care and the training of culturally competence nurses in caring for older adults living with COPD.

## Conclusion

The knowledge gained from this study can help health professionals, including nurses, and family caregivers in regions or countries with Muslim minorities. Understanding the barriers and facilitators associated with caring for older adults with COPD during transitional care should be emphasized. Nurses and hospital management should improve discharge planning for older people with COPD by addressing some issues. This includes dyspnea symptom management at home, improving the continuity of care for older adults with COPD, and improving the cultural competency of caring for Thai Muslim.

### Electronic supplementary material

Below is the link to the electronic supplementary material.

Supplementary Material 1

Supplementary Material 2

## Data Availability

The research data are not publicly available due to privacy and ethical restrictions (participant consent forms stated that data will only be available to the principal investigator and the research team.

## Abbreviations

COPD: Chronic obstructive pulmonary disease
COVID-19: Coronavirus disease 2019
WHO: World Health Organization
ED: Emergency department
FG: Family caregivers
FGDs: Focus group discussions
D-METHOD D: Disease, M: Medication, E: Environment, T: Treatment, H: Health, O: Outpatient appointment, D: Diet
COC: Continuum of Care
PPE: Personal protective equipment
