# Supplementary material for: Perspectives of family caregivers and nurses on hospital discharge transitional care for Muslim older adults living with COPD: a qualitative study
Source: BMC Nurs. 2024 Apr 24;23:273. doi: 10.1186/s12912-024-01943-8 (PMC11044287; doi:10.1186/s12912-024-01943-8)
Supplement: Supplementary file 1 — Supplementary Material 1 [file 12912_2024_1943_MOESM1_ESM.docx]

**The instruments used for research**

**The instruments used for data collection in this study consisted of two main parts regarding to research and development phase**

**Part 1** phase of brainstorming and understanding the barriers of caring older adults with COPD by using focus group discussion and semi-structured interview

**Instruction** This instrument used for phase 1 of data collection in order to brainstorm as well as understanding the situation, needs, in the area regarding to emergency health management in hospital transition to reduce ED visit for older adults with COPD in perspectives of health care receivers and health care providers for proper analyzing and developing program.

**The targeted sample consisted of: 1) health care receivers (family caregiver), and 2) health care providers**

**1.1 Family caregivers of older adults with COPD**

Personal data information

1. Relationship with older adults with COPD………………………….
2. Gender………………………………………………………………..
3. Age……………………………………………………………………..
4. Marital status………………………………………………………….
5. The highest level of education………………………………………….
6. Occupation…………………………………………………………….
7. Role of caring for older adults with COPD……………………………..
8. The amount of time caring for older adults per day…………………………
9. The role of caring other persons………………………………………………
10. Experience of caring in emergency situation or calling 1669……………………………………………………………………………

Question guidelines for each family caregiver of older adults with COPD

1. How older adults with COPD caring for themselves at home?
2. How do you facilitate or assist in caring for older adults with COPD at home?
3. Any risk factors can lead for dyspnea in older adults with COPD and how you dealt with them?
4. Do you have any experiences in caring for older adults with COPD during dyspnea? How do you have encountered with this situation? How were the results and when do you need to bring older adults with COPD visiting ED?
5. What is the core knowledge that need for caring of older adults with COPD at home?
6. What are the activities that important for dyspnea symptom management at home for older adults with COPD?
7. Any barriers of caring for older adults with COPD at home? How you dealt with that barrier?
8. Do you need any facilitators in caring for older adults with COPD at home to prevent for ED visit in the future?
9. Do you feel satisfy with caring by health care providers, what should it be in caring for older adults with COPD in previous experience?
10. If somebody asking for advice or recommendation from you regarding to how to prevent for acute exacerbation in older adults with COPD, what you will give them an advice?

**1.2 Health care providers consisted of nurses**

**Personal data**

The questionnaire code number………………………………………

Date of interview…………Month………………………Year…………….

1. The workplace…………………………………………………………………..
2. Gender…………………………………………………………………………..
3. Age………………………………………………………………………
4. Marital status…………………………………………………………….
5. Religion…………………………………………………………………
6. The role of work………………………………………………………….
7. Numbers of older adults with COPD have responsible with……………
8. Years of experience of caring for older adults with COPD……………..
9. Training of caring for older adults with COPD………………………..

**Question guidelines for focus group discussion**

1. From your experience, what are the risk factors contributing to develop acute exacerbation resulting to visit ED?
2. How did you perform of caring of older adults with COPD prior and post discharge from hospital?
3. What is the information/knowledge that you promote for older adults with COPD to enhance symptom management ability at home? Do you think are there any information/knowledge that were already sufficient or still insufficient?
4. Do you have any experience dealing with dyspnea in older adults with COPD? What are the facilitator of dyspnea symptoms management at home that can prevent of ED visit in this population?
5. Are there any activities in the community to prevent ED visits in older adults with COPD?
6. Are there any activities that need to be revised regards needs, living, belief, culture, society, and environment of older adults with COPD?
7. Do you think the exciting continuity of care system can be utilized and fitted with older adults with COPD in order to reduce ED visit?
8. What self-care activities in the community that older adults with COPD can utilize and adapt in own self-care to reduce ED visit?
9. Any barriers or difficulty in order to reduce ED visit in older adults with COPD?
10. Any facilitators that you need in emergency health management program in order to reduce ED visit in older adults with COPD?
