# Supplementary figures and images for: Perspectives of family caregivers and nurses on hospital discharge transitional care for Muslim older adults living with COPD: a qualitative study
Source: BMC Nurs. 2024 Apr 24;23:273. doi: 10.1186/s12912-024-01943-8 (PMC11044287; doi:10.1186/s12912-024-01943-8)

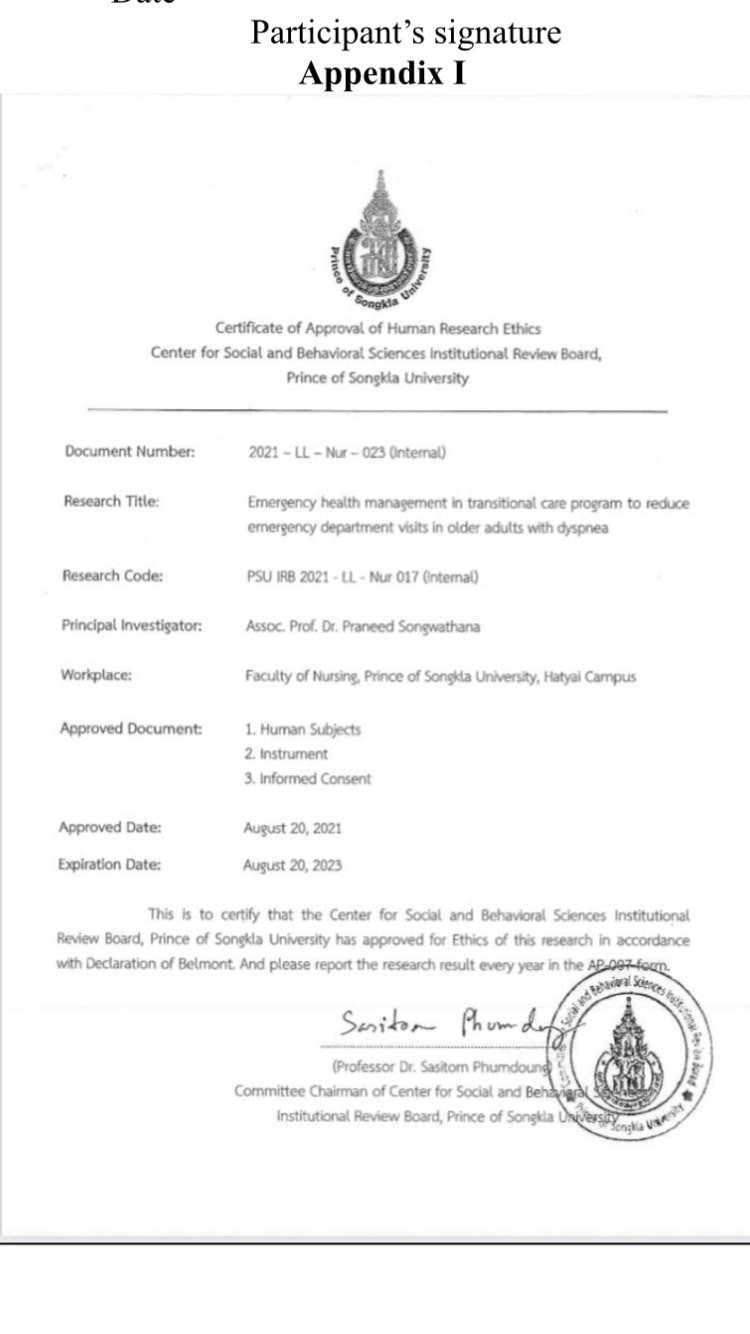

Supplement: Supplementary file 2 — Supplementary Material 2 [file 12912_2024_1943_MOESM2_ESM.jpg]
